# *Pneumocystis jirovecii* among patients with cystic fibrosis and their household members

**DOI:** 10.1093/mmy/myab010

**Published:** 2021-03-10

**Authors:** Ruben Morilla, Francisco J Medrano, Ana Calzada, Esther Quintana, Elena Campano, Vicente Friaza, Enrique J Calderón, Carmen de la Horra

**Affiliations:** Instituto de Biomedicina de Sevilla (Hospital Universitario Virgen del Rocío/ CSIC/ Universidad de Sevilla), 41013 Seville, Spain; Centro de Investigación Biomédica en Red de Epidemiología y Salud Pública (CIBERESP), Instituto de Salud Carlos III, Madrid, Spain; Department of Internal Medicine, Hospital Universitario Virgen del Rocío, 41013 Seville, Spain; Department of Nursing, Universidad de Sevilla, Seville, Spain; Instituto de Biomedicina de Sevilla (Hospital Universitario Virgen del Rocío/ CSIC/ Universidad de Sevilla), 41013 Seville, Spain; Centro de Investigación Biomédica en Red de Epidemiología y Salud Pública (CIBERESP), Instituto de Salud Carlos III, Madrid, Spain; Department of Internal Medicine, Hospital Universitario Virgen del Rocío, 41013 Seville, Spain; Department of Medicine, Universidad de Sevilla, 41013 Seville, Spain; Hospital Virgen de las Montañas de Villamartín, 11650 Cadiz, Spain; Unidad Médico-Quirúrgica de Enfermedades Respiratorias, Instituto de Biomedicina de Sevilla (IBiS), Hospital Universitario Virgen del Rocío/Universidad de Sevilla, 41013 Seville, Spain; Centro de Investigación Biomédica en Red de Enfermedades Respiratorias (CIBERES), Instituto de Salud Carlos III, Madrid, Spain; Instituto de Biomedicina de Sevilla (Hospital Universitario Virgen del Rocío/ CSIC/ Universidad de Sevilla), 41013 Seville, Spain; Instituto de Biomedicina de Sevilla (Hospital Universitario Virgen del Rocío/ CSIC/ Universidad de Sevilla), 41013 Seville, Spain; Centro de Investigación Biomédica en Red de Epidemiología y Salud Pública (CIBERESP), Instituto de Salud Carlos III, Madrid, Spain; Instituto de Biomedicina de Sevilla (Hospital Universitario Virgen del Rocío/ CSIC/ Universidad de Sevilla), 41013 Seville, Spain; Centro de Investigación Biomédica en Red de Epidemiología y Salud Pública (CIBERESP), Instituto de Salud Carlos III, Madrid, Spain; Department of Internal Medicine, Hospital Universitario Virgen del Rocío, 41013 Seville, Spain; Department of Medicine, Universidad de Sevilla, 41013 Seville, Spain; Instituto de Biomedicina de Sevilla (Hospital Universitario Virgen del Rocío/ CSIC/ Universidad de Sevilla), 41013 Seville, Spain; Centro de Investigación Biomédica en Red de Epidemiología y Salud Pública (CIBERESP), Instituto de Salud Carlos III, Madrid, Spain; Department of Microbiology and Parasitology, School of Pharmacy, University of Seville, 41013 Seville, Spain

**Keywords:** *Pneumocystis jirovecii*, disease transmission, cystic fibrosis, polymerase chain reaction

## Abstract

We conducted a pilot study of patients with cystic fibrosis (CF) to assess intra-family transmission of *P. jirovecii* and compare it with data on other prevalent pathogens such as *P. aeruginosa* and *S. pneumoniae*, in which respiratory transmission has already been documented. Oral swab samples from 10 patients with CF and 15 household members were collected at baseline and 2 weeks later. *P. aeruginosa* and *S. pneumoniae* were assessed using standardized culture methods and PCR, and *P. jirovecii* was assessed using real and nested PCR, genotyping the positive samples by direct sequencing. *P. aeruginosa* cultures were positive for 7/10 (70%) of patients with CF at baseline and was identified by PCR in 8/10 (80%) of cases at baseline and 2 weeks later. *S. pneumoniae* cultures were negative for all patients, but the microorganism was identified by PCR in two cases. *P. jirovecii* was detected by real time and nested PCR in 5/10 (50%) of the patients at the two time points. In the household members, *P. aeruginosa* and *P. jirovecii* were identified in 7/15 (46.7%), and *S. pneumoniae* was identified in 8/15 (53,3%). The concordance of positive or negative pairs of patients with CF and their household members was 33.3% (5/15) for *P. aeruginosa*, 46.7% (7/15) for *S. pneumonia* and 93.3% (14/15) for *P. jirovecii*. The concordance for *P. jirovecii* genotypes among five pairs with available genotype was 100%. This study suggests for the first time the possible transmission of *Pneumocystis* in the home of patients with CF, indicating that patients and their household members are reservoirs and possible sources of infection.

**Lay Summary:**

This study suggests for the first time the possible transmission of *Pneumocystis* in the family environment of patients with cystic fibrosis, indicating that patients and their household members are reservoirs and possible sources of this infection.

## Introduction

*Pneumocystis* pneumonia (PCP) is a life-threatening fungal infection that affects immunosuppressed individuals worldwide and is currently an AIDS-defining opportunistic infection in patients with HIV.^1^ Immunocompromised patients without HIV are also susceptible to infection, which has led to an update of the guidelines for prophylaxis for PCP in these individuals.^2,3^

Studies on the epidemiology of *Pneumocystis jirovecii* have revealed the presence of this microorganism in both apparently healthy and immunocompetent individuals without overt clinical manifestations of PCP, indicating colonization.^4^ This phenomenon has been observed through DNA amplification techniques performed on respiratory samples such as bronchoalveolar lavage (gold standard), sputum (induced or not), oral wash and lung tissue.^1,4–6^

The colonization status has gained importance for two reasons: (1) its proposed association with a negative prognosis for chronic pulmonary diseases, given that it induces a proinflammatory response and increased mucus production^7,8^; (2) its postulated important role in disseminating this microorganism, given that colonized individuals can act as reservoirs and possible sources of infection.^1,4^

*P. jirovecii* has been identified in patients with various chronic pulmonary diseases, including chronic obstructive pulmonary disease, interstitial lung diseases and cystic fibrosis (CF),^4^ a genetic disease of autosomal recessive inheritance that mainly affects the lungs but can also secondarily affect the pancreas, liver and intestine, leading to the accumulation of highly tenacious mucus in these areas. CF is one of the most common types of chronic lung disease in children and young adults and is a life-threatening condition.^9^ However, the major contributing factor to the development of pulmonary disease in patients with CF is persistent infection by opportunistic pathogens, where *Staphylococcus aureus* and mucoid *Pseudomonas aeruginosa* continue to be the key pulmonary pathogens.^10^

The presence of *P. jirovecii* in these patients has been evaluated in several countries, with varying reported prevalence^11^, from low colonization rates of 2.5% in Brittany (France)^12^ and 7.4% in Munich (Germany)^13^ to higher rates of 21.5% in Seville (Spain)^14^ and 38.2% in Porto Alegre (Brazil).^15^ A number of factors proposed to explain this variability include methodological disparities (type of sample or DNA amplification protocol), patient age and previous prophylaxis with cotrimoxazole.^12–16^

A recent study reported a possible association between *Pneumocystis* colonization and acute exacerbations in patients with CF, suggesting a role for this fungus in the pathophysiology of CF.^16^ These findings emphasize the importance of understanding the mechanisms of transmission and sources of infection from which patients with CF can be infected by *Pneumocystis*. Experimental animal models^17^ and nosocomial outbreaks of PCP^18^ suggest airborne transmission as the most plausible mechanism. Our group reported molecular evidence of *Pneumocystis* transmission between an infant and her grandparents living in the same household.^19^

The aim of our study was to evaluate the possible intra-family transmission of *P. jirovecii* and to compare it with data related to other prevalent pathogens in patients with CF such as *P. aeruginosa* and *S. pneumoniae*, in which respiratory transmission in the family environment has already been documented.^20,21^

## Methods

We conducted a pilot observational study to evaluate the presence of the non-cultivable fungus *P. jirovecii* by molecular techniques (polymerase chain reaction [PCR]) at baseline and after 2 weeks. The presence of *Pseudomonas aeruginosa* and *Streptococcus pneumoniae*, commonly found in the oropharynx of patients with CF, was evaluated by standardized culture methods and PCR, also at baseline and after 2 weeks.

Ten patients with CF were recruited consecutively from November 1, 2015 in a reference outpatient clinic for cystic fibrosis of a tertiary hospital (Hospital Universitario Virgen del Rocío, Seville, Spain) that, fulfilling the inclusion criteria, voluntarily agreed to participate in the study. The inclusion criteria were: (i) over 10-year-old, (ii) diagnosis of CF defined as a sweat chloride value ≥ 60 mmol/l and clinical features consistent with CF or a positive family history of CF and confirmed in all cases by identification of two disease-causing CF transmembrane conductance regulator gene mutations; (iii) possibility of obtaining a respiratory sample of conventional or induced sputum and also capable of gargling to obtain an oropharyngeal lavage, (iv) absence of lung transplantation and/or immunosuppressive therapy.

All the household members who (at least the last 6 months) accompanied the patient during the visit to the clinic were invited to participate in the study. Furthermore, they should be able to return after 14 days (scheduled visit according to clinical protocol for these patients) for a second sample collection. All patients and their parents or legal guardians, in the case of minors, signed a written informed consent.

Samples from patients with CF and household members were collected from the oral cavity by a specific swab for bacteria and fungi and were kept frozen until received by the laboratory. Sputum samples from patients with CF were also collected in duplicate. One of the sputum aliquots from the patients was also sent to the Microbiology Department for bacteria culture and the other was frozen at –20ºC for further study.

Genomic DNA was extracted using the Nucleo Spin Tissue commercial kit (Machenary Nagel, GmbH & Co. KG, Germany) after sample digestion at 56ºC using 14 mg/ml proteinase K, 2 mM ethylenediaminetetraacetic acid and 10% sodium dodecyl sulfate. The solution was shaken for at least 4 h.

To identify DNA from *P. aeruginosa*, we followed a previously described PCR protocol using the consensus primers PA01-A (CAggTCggAgCTgTCgTACTC) and PAS01-S (ACCCgAACgCAggCTATg), which amplify a 113 bp fragment.^22^ The PCR reaction took place with 2-μl DNA in 23 μl of a PCR reaction mix (200 μM of each nucleotide triphosphate; 20 pmol of each primer; 2.5 U Taq- polymerase and 2.5 μl of 10× Taq Buffer), 1.2 mM MgCl_2_, and sterile water. For the hot start, the PCR mixture was heated to 95ºC for 5 min. The program consisted of 40 cycles of the following: denaturing at 92ºC for 60 s, primer annealing 58ºC for 60 s and extension at 72ºC for 2 min. Last, a step at 72ºC was performed for 5 min for the final extension.

To identify *S. pneumoniae*, we employed the previously described PCR protocol *LytA* target gen, which encodes a region of 101 bp using the primers LytA-F ACgCAATCTAgCAgATgAAgC and LytA-R TgTTTggTTggTTATTCgTgC.^23^ The reaction mix was prepared to a final volume of 20 μl and included 200 μM of each nucleotide triphosphate; 20 pmol of each primer; 2 U Taq- polymerase and 2 μl of 10× Taq Buffer, 1 mM MgCl_2_, sterile water and a 2-μl DNA template. The PCR reaction was performed in a single protocol with a hot start step at 95ºC for 5 min, 40 cycles (denaturing at 92ºC for 60 s, annealing at 57ºC for 60 s and extension at 72ºC for 2 min) and a final extension step at 72ºC for 5 min.

Identification of *P. jirovecii* colonization was performed by analyzing the samples of the gene encoding the mtLSU-rRNA, with primers pAZ102-X and pAZ102-Y. We adapted the method previously described to a real-time PCR setting,^24^ using a LightCycler 1.5 (Roche Molecular Biochemicals, Mannheim, Germany). The PCR mixture contained 20 pmol of both primers pAZ102-X and pAZ102-Y, as well as 10 to 40-ng DNA, and amplified using LightCycler® DNA Master SYBR Green I (Roche Molecular Biochemicals) to a final concentration of 2.5 mM Mg^2+^. After an initial denaturation step at 95°C for 10 min, amplification was performed with 40 cycles of denaturation (95°C for 5 s), annealing (55°C for 15 s) and extension (72°C for 10 s). The PCR product was detected using SYBR Green I dye, which strongly increases its fluorescence after binding to double-stranded DNA. In all positive samples by real-time PCR, a second nested-PCR was performed in another aliquot with the same primers to characterize the genotype of the PCR product.

To prevent contamination, pipettes with filters were used for all manipulations. DNA extraction and preparation of the reaction mixture were performed in two different rooms using separate laminar flow hoods. A 256-bp amplification product from *Pneumocystis* cloned into the pGEM T-EASY vector (Promega, Madison, WI) was run simultaneously with samples as positive controls. We employed autoclaved water in the PCR mixture as a negative control.

The genotype characterization of *P. jirovecii* was performed according to a previously described protocol.^24^ Briefly, the nested-PCR products from mtLSU-rRNA were purified using Sephacryl S-400 columns (Amersham Pharmacia Biotech AB) and reamplified using ABI Prism dRhodamine Terminator Cycle Sequencing Ready Reaction Kit (PE Applied Biosystems, Foster City, CA). For each reaction, 5 μl of PCR product, 4 μl of terminator-ready reaction mix and 3 pmol of primer were then added. The extension products were purified using an ethanol precipitation procedure to remove excess dye terminators. Each sample pellet was resuspended in 12.5 μl of template suppression reagent and heated at 95ºC for 3 min for denaturing. The electrophoresis was performed in the ABI prism 310 sequencer (PE Applied Biosystems, Foster City, CA) according to the manufacturer's recommendations. The sequenced DNA fragments were analyzed using Sequence Navigator v.1.0.1 (PE Applied Biosystems, Foster City, CA).

Regarding molecular tests, a patient with CF was considered positive for a microorganism (*P. aeruginosa, S. pneumoniae* or *P. jirovecii*) when the DNA of the pathogen was amplified in both swab and sputum samples. In household members, only DNA swab amplification was considered positive.

The study was reviewed and approved by the Ethics Committee of Virgen del Rocio University Hospital (Seville, Spain), The participants provided their written informed consent to participate in this study. The descriptive analysis to show the clinical and sociodemographic profile of the patients with CF was performed using the IBM SPSS software package (version 24, SPSS Inc., Chicago, IL, USA).

## Results

The study included 10 patients with cystic fibrosis and 15 household members. Table 1 shows the patients’ demographic and clinical data. During the 6 months prior to the study, 5/10 (50%) of the patients were treated with inhaled corticosteroids and 9/10 (90%) took antibiotics. None of them smoked.

**Table 1.** Clinical and epidemiological features of patients with cystic fibrosis.

Variable	
Age, years (mean ± SD)	19.7 ± 7.42
Body-mass index, kg/m^2^ (mean ± SD)	17.73 ± 3.03
FEV1 (mean ± SD)	60.77 ± 20.87
Sex, n (% males)	2 (20%)
Exocrine pancreatic insufficiency, n (%)	6 (60%)
Treatment with inhaled corticosteroids*, n (%)	5 (50%)
Antibiotherapy**, n (%)	9 (90%)

SD, standard deviation; FEV1, forced expiratory volume in 1 second; * during the 6 months prior to the study; ** any antibiotic during the 6 months prior to the study (none received cotrimoxazol or levofloxacin).

### Identification of microorganisms in patients with cystic fibrosis

*P. aeruginosa* conventional cultures were positive at baseline in 70% of the samples and in 8/10 (80%) of the patients with CF by PCR at baseline and 2 weeks later. *S. pneumonia* cultures were negative for all patients, but the microorganism was identified by PCR in two of the patients with CF (CF-05 and CF-09). DNA amplification of *P. aeruginosa* was observed in one patient (CF-10) with a negative culture.

*P. jirovecii* was detected by real-time PCR in 5/10 (50%) of patients at the two evaluated time-points. All positive samples in real-time PCR were amplified in the nested PCR. Table 2 summarizes the data, showing the results for the various approaches. None of the patients had a previous history of PCP.

**Table 2.** Microorganisms identified in patients with CF and their household members.

		*P. aeruginosa*			*S. pneumoniae*			*P. jirovecii*			
		Patient with CF			Patient with CF			Patient with CF		Household members	
				Household			Household				
Patient code	Relative code	Culture	PCR 0 2w	members	Culture	PCR 0 2w	members	PCR 0 2w	Genotype	PCR	Genotype
CF-01	R-301(M)	**+**	**+ +**	**-**	**-**	**- -**	**+**	**+ +**	3	+	3
	R-315(F)			**-**			**+**			+	3
CF-02	R-302(H)	**+**	**+ +**	**+**	**-**	**- -**	**-**	**- -**	-	-	-
CF-03	R-303(M)	**-**	**- -**	**+**	**-**	**- -**	**-**	**+ +**	1	+	1
CF-04	R-304(F)	**+**	**+ +**	**-**	**-**	**- -**	**+**	**+ +**	3	+	NS
	R-305(H)			**-**			**+**			+	3
CF-05	R-306(M)	**+**	**+ +**	**+**	**-**	**+ +**	**+**	**- -**	-	-	-
CF-06	R-307(F)	**+**	**+ +**	**+**	**-**	**- -**	**-**	**+ +**	3	+	3
CF-07	R-308(F)	**+**	**+ +**	**-**	**-**	**- -**	**+**	**+ +**	1	+	NS
	R-309(M)			**-**			**+**			**-**	**-**
CF-08	R-310(M)	**+**	**+ +**	**+**	**-**	**- -**	**+**	**- -**	**-**	**-**	**-**
	R-311(A)			**-**			**-**			**-**	**-**
	R-312(F)			**+**			**-**			**-**	**-**
CF-09	R-313(M)	**-**	**- -**	**+**	**-**	**+** ND	**-**	**- -**	**-**	**-**	**-**
CF-10	R-314(H)	**-**	**+ +**	**-**	**-**	**- -**	**-**	**- -**	**-**	**-**	**-**

(M), mother; (F), father; (H), husband; (A), aunt; CF, Cystic fibrosis; 2 w, week 2; ND, non-detected; NS, non-sequenced.

### Microorganisms identified in household members of patients with cystic fibrosis

*P. aeruginosa* and *P. jirovecii* were identified by PCR in 7/15 (46.7%) of the household members of the patients with CF. The amplification of mtLSU locus in samples R-304 and R-308 was weak and after their purification there was not enough material to proceed with their sequencing. The most frequent microorganism found was *S. pneumoniae* at 8/15 (66.7%) (Table 2).

### Microorganisms in the cystic fibrosis family environment

The data obtained by PCR for *P. aeruginosa* showed matching results in 5/15 (33.3%) of the pairs of familial cases (patient/relative both positives or patient/relative both negatives), and the remaining were discordant (patient positive and relative negative or patient negative and relative positive). The analysis of concordance of positive or negative pairs was 46.7% (7/15) for *S. pneumoniae*.

However, the results obtained for *P. jirovecii* colonization showed a concordance of positive or negative pairs of 93.3% (14/15). Among the colonized participants, *P. jirovecii* was sequenced at mt LSU rRNA in 10 of the 12 positive samples. Genotype 3 (85C, 248C) was isolated in seven, and genotype 1 (45T, 248C) was isolated in three. As shown in Table 2, the concordance among genotypes at mt LSU for *P. jirovecii* was 100% in the five pairs of patients with CF and their household members.

## Discussion

The current study provides new evidence of the importance of the transmission and sources of infection of *Pneumocystis*, in this case, in patients with CF. In fact, this is the first pilot study to evaluate the contemporaneous colonization of patients and household members, showing a high rate of concordance, not only in *Pneumocystis* colonization but also in the presence of other microorganisms typically associated with these patients.

Patients with CF are frequently colonized or infected by bacteria, meriting investigation that these patients’ lungs might also represent a niche for *Pneumocystis. P. aeruginosa* is the microorganism that most frequently colonizes patients with CF,^25^ however, studies on *P. aeruginosa* transmission among patients with CF have shown conflicting results.^26,27^ In our study, an 80% (8/10) colonization rate confirms the high rate of exposure to this microorganism.

*S. pneumoniae*, whose intra-family transmission has been previously demonstrated,^28^ was only identified in 2/10 (20%) of patients with CF through molecular techniques (none through culture methods) and was present in 8/15 (53.3%) of household contacts. These findings could be explained because vaccination is not currently recommended in individuals younger than 65 years,^29^ while patients with cystic fibrosis remain vaccinated because they are people at risk.

The prevalence of *Pneumocystis* colonization in patients with CF ranges from 2.5 to 38.2% depending on the geographic location,^12–15^ however, the prevalence in our study was 50%, which is higher than that we previously reported.^14^ This higher prevalence was probably due to the small sample size rather than possible contamination problems, as the appropriate safety measures were taken and positive results were confirmed in different samples in each case.

This microorganism was present in 7/15 (46.7%) of household members of patients with CF, a much higher percentage than the 13% (95% CI: 7.9-18%) reported in the general population of our area.^30^

The role of patients with CF as a reservoir for the organism and as a possible source of infection for susceptible individuals has been previously reported.^4^ This study extends this hypothesis to their household members, reporting a rate of colonization that agrees with the colonization status of the patient with CF with whom they live. We have argued that colonization status is not a permanent status but rather consists of various cycles of colonization and clearance of the microorganism, with an as yet unclear duration of these cycles.^31–32^

Recent studies have reported the airborne transmission of *Pneumocystis* in hospitals by identifying this fungus in the air of rooms of colonized patients,^33^ molecular evidence of transmission of this fungus from patients to healthcare workers,^32–34^ and transmission maps created to investigate the nosocomial transmission of this microorganism.^35^

Taken together, these findings on transmission modes and transient colonization suggest mechanisms by which *Pneumocystis* remains viable until contact with a susceptible individual.

*Pneumocystis* transmission within families has also been documented, but data are still scarce.^36–40^ This study shows matching genotypes of *Pneumocystis* among patients and their household members, with only genotypes 3 and 1 of the mtLSU locus found to be the predominant genotypes in previous studies from Spain^14,31^ and Brazil.^15^ Patients with a positive PCR at baseline (5/10) remained positive 2 weeks later. These findings indicate a potential common source of transmission among individuals who live in the same household.

The main limitation of the study is the sample size, although this constraint and some of its consequences have already been discussed. We design a pilot study with few families and only two evaluations over time due to the high cost derived from the number of diagnostic test needed and the difficulty of recruiting household members for following-up over time. The absence of a control group of families of individuals without CF makes it impossible to compare whether the phenomenon is more frequent in one type of family or another. We avoided potential false-positives by adopting stringent precautionary measures and by examining the PCR signal with two types of PCR in different aliquots of the samples and different samples in the case of CF patients.

On the other hand, the concordance of genotypes in members of a family does not exclude the presence of a common source of infection, encouraging to develop more ambitious studies to confirm the hypothesis of the possible intrafamiliar transmission of *Pneumocystis* infection in patients with CF.

The *Pneumocystis* colonization status of patients with CF could be associated with acute pulmonary exacerbations.^16^ Therefore, our results could have not only epidemiological implications but also clinical consequences for these patients. The possibility that *Pneumocystis* colonization could be linked to negative health outcomes for patients with CF highlights the need to evaluate the possibilities for preventive prophylaxis or preventive measures.

In conclusion, the current study suggests for the first time the possible transmission of *Pneumocystis* in the family environment of patients with CF, indicating patients and their household members as reservoirs and possible sources of the infection. Thus, patients with CF could be exposed to this microorganism, which might affect their health and the course of the disease.
